# Elevated IL-6, IL-10, and IFN-γ levels in fatal elephant endotheliotropic herpesvirus – hemorrhagic disease cases suggest an excessive proinflammatory cytokine response contributes to pathogenesis

**DOI:** 10.3389/fimmu.2025.1645752

**Published:** 2025-10-27

**Authors:** Tabitha E. Hoornweg, Willem Schaftenaar, Jooske IJzer, Myrna M. P. Mulder, Mariska Lugtenburg, Anne van Beest, Cornelis A. M. de Haan, Victor P. M. G. Rutten

**Affiliations:** ^1^ Section Immunology, Division Infectious Diseases and Immunology, Department of Biomolecular Health Sciences, Faculty of Veterinary Medicine, Utrecht University, Utrecht, Netherlands; ^2^ Section Virology, Division Infectious Diseases and Immunology, Department of Biomolecular Health Sciences, Faculty of Veterinary Medicine, Utrecht University, Utrecht, Netherlands; ^3^ European Association of Zoos and Aquaria (EAZA) Elephant Taxon Advisory Group (TAG), Rotterdam Zoo, Rotterdam, Netherlands; ^4^ Division Pathology, Department of Biomolecular Health Sciences, Utrecht University, Faculty of Veterinary Medicine, Utrecht, Netherlands; ^5^ Department of Veterinary Tropical Diseases, Faculty of Veterinary Science, University of Pretoria, Onderstepoort, South Africa

**Keywords:** EEHV, EEHV-HD, elephant, cytokine, immunopathogenesis, IL-6, IL-10, IFN-γ

## Abstract

**Introduction:**

Hemorrhagic disease developed as a consequence of an EEHV infection (EEHV-HD) is the leading cause of death of young Asian elephants in Zoos worldwide and also affects elephants in range countries. Although a cytokine storm has long been suggested to underlie disease pathogenesis, there is little evidence and the role of cytokines in EEHV-HD pathogenesis remains unclear to date.

**Methods:**

In the current study, we compared mRNA levels of eight different cytokines between blood and tissue samples of EEHV-HD cases (n=11) and controls (n=12) in order to determine whether cytokines may contribute to EEHV-HD pathogenesis.

**Results:**

We show the presence of significantly elevated mRNA levels of IFN-γ, IL-6 and IL-10, cytokines typically associated with cytokine storms, in blood or tissues with high viral loads (heart and liver) of EEHV-HD cases. Comparable cytokine inductions were not observed in tissues with lower viral loads (tongue, lung and kidney), indicating an association between viral replication and cytokine induction, and suggesting damage observed in these tissues is likely collateral.

**Discussion:**

In conjunction with pathological findings, including acute systemic inflammation and multiple organ dysfunction, we propose that a pathogen-induced cytokine storm indeed underlies EEHV-HD pathogenesis, which would support investigation into the use of anti-inflammatory therapies to control disease.

## Introduction

Elephant endotheliotropic herpesviruses are a group of seven herpesviruses (EEHV1-7) that either infect Asian (*Elephas maximus*) or African elephants (*Loxodonta* species) (1). All adult elephants, both in zoos and range countries, are seropositive for multiple EEHVs, suggesting that all elephants get infected with these viruses during their lives (2–5). In line with other herpesviruses, EEHVs establish lifelong latent infections, with occasional reactivations, mostly without causing clinical signs (6). Young elephants, however, may develop an acute, highly fatal hemorrhagic disease, known as EEHV hemorrhagic disease (EEHV-HD), upon infection with an EEHV (sub)species (1). Disease is typically seen in elephants between one and ten years of age that have low to non-detectable antibodies levels to the (sub)species causing infection (2). Although all EEHVs may cause fatal EEHV-HD, subspecies EEHV1A, infecting Asian elephants, has been responsible for the vast majority of fatalities to date (1, 2). Although EEHV-HD was long thought to affect young Asian elephants solely, multiple young African elephants born in Western zoos have recently succumbed to EEHV-HD as well (7–12).

Young elephants affected by EEHV-HD initially present with non-specific clinical signs including lethargy, anorexia and lameness, which may quickly progress into more specific clinical signs such as cyanosis of the tongue and oedema of the face, neck, and front legs (1, 13). Eighty to eighty-five percent (80-85%) of the affected animals die within days after the onset of disease as a result of widespread internal hemorrhages, including extensive transmural myocardial hemorrhages, inflicting severe myocardial damage, and subsequent cardiac failure (14–16). The virus infects a wide array of tissues in affected animals, with the highest viral loads generally detected in heart, liver, and tongue (17, 18). In all these tissues the virus infects the endothelial cells lining the microvasculature, causing endothelial damage and pathogenic alterations of the coagulation cascade (14–16, 19). Several recent studies suggest that disseminated intravascular coagulation (DIC), an acquired syndrome in which accelerated clotting within blood vessels leads to massive consumption of platelets and eventually causes uncontrollable bleeding (20), contributes to disease severity (14–16).

DIC may be triggered by many factors, such as severe systemic infections, major tissue destruction, and strong immunological reactivity (20). In analogy to viral hemorrhagic fevers in humans, caused by viruses such as Ebola virus (EBOV), Lassa virus (LASV) and Dengue virus (DENV) (21), it has been proposed that DIC and the resulting severe clinical signs observed during EEHV-HD are triggered by an uncontrolled systemic pro-inflammatory cytokine activation, known as a ‘cytokine storm’ (14, 15). During cytokine storms elevated levels of cytokines such as interferon-γ (IFN-γ), interleukin (IL)-1, IL-6, IL-18, and tumor necrosis factor-α (TNF-α) are often measured, and these cytokines are thought to have central roles in immunopathogenesis (22). Additionally, elevated levels of the immunomodulatory cytokine IL-10 are often observed, most likely reflecting a regulatory response to the abundant induction of the proinflammatory cytokines (22). The exact cytokines found to be upregulated however differ between types of cytokine storms and depend on the stage of inflammation (22–26).

Since cytokines play crucial roles in the protective immune response against pathogens, it is often difficult to distinguish a cytokine storm from a proportional, and thereby normal, immune response during the course of an infection (22). Therefore, three criteria were proposed to identify a cytokine storm in the context of infection, namely (i) detection of elevated circulating cytokine levels, (ii) presence of acute systemic inflammatory symptoms, and (iii) secondary organ dysfunction beyond what could be attributed to a normal response to a pathogen (22). While three independent studies reported the presence of acute systemic inflammation and secondary organ dysfunction in EEHV-HD fatalities (14–16), so far only two studies tried to assess the cytokine response during EEHV-HD, with conflicting conclusions (15, 27). Guntawang and coworkers concluded that a cytokine storm indeed occurs during EEHV-HD since upregulation of several cytokines was detected in heart, liver and lung samples of EEHV-HD fatalities. However, the cytokines for which significantly elevated expression levels were reported and their extent of induction varied widely between the tissue samples examined (15). On the other hand, Edwards and coworkers suggested that an inadequate immune response to the virus may be underlying disease progression, since only low levels of TNF-α and IL-2 could be detected in serum of EEHV-HD fatalities (27).

In view of these conflicting conclusions, in the current study we measured mRNA levels of eight different cytokines (IL-2, IL-4, IL-6, IL-10, IL-12, IFN-γ, transforming growth factor-β (TGF-β), and TNF-α) in blood and tissue samples of 23 elephants and compared these between EEHV-HD cases (n=11) and controls (n=12), aiming to elucidate whether an excessive cytokine response may indeed be correlated with and hence contributes to EEHV-HD pathogenesis.

## Materials and methods

### Origin, handling, and storage of elephant samples

Samples of 23 elephants kept under human care in fourteen different European Zoos were included in the study. An overview of species, age, health status and the available blood and tissue samples of each elephant is shown in **Table 1**.

**Table 1:** Overview of the study samples.

#	Species	Age at sampling (y)	Health status when last sampled	EEHV-HD	Cause of death	Sample types included	
						Blood	Tissue
1	EM	0,8 - 1,5	Healthy	-	-	×^a(3),b(1)^	
2	EM	1,3	Healthy	-	-	×^a^	
3	EM	4,2	Healthy	-	-	×^a^	
4	EM	8,2	Healthy	-	-	×^a^	
5	EM	10,6	Healthy	-	-	×^a^	
6	EM	~22	Healthy	-	-	×^b(2)^	
7	EM	1,7	Ill	EEHV1A	Survived	×^a(3)^	
8	EM	4,9	Ill	EEHV1B	Survived	×^a(2)^	
9	EM	1,1	Died	EEHV1A	EEHV-HD		×^d,e^
10	EM	1,4	Died	EEHV1A	EEHV-HD		×^d,g^
11	EM	2,2	Died	EEHV1A	EEHV-HD	×^a^	×^c-g^
12	EM	2,6	Died	EEHV1A	EEHV-HD		×^c-g^
13	EM	2,9	Died	EEHV1A	EEHV-HD	×^a^	
14	EM	3,0	Died	EEHV1A	EEHV-HD	×^b(2)^	×^c-g^
15	EM	2,5 - 3,6	Died	EEHV1B	EEHV-HD	×^a(1),b(4)^	×^c-g^
16	EM	4,1	Died	EEHV5	EEHV-HD	×^a^	×^c-g^
17	EM	0,1	Died	-	Other cause		×^d^
18	EM	0,9	Died	-	Other cause		×^c-g^
19	EM	9,1	Died	-	Other cause		×^c-g^
20	EM	~40	Died	-	Other cause		×^c-g^
21	EM	~53	Died	-	Other cause		×^c-g^
22	LA	2,0	Died	EEHV6	EEHV-HD		×^c,d,f,g^
23	LA	0	Died	-	Other cause		×^c,d,f,g^

Per individual animal, its species, age at sampling (in years), health status when last sampled, the subspecies causing EEHV-HD (if applicable), cause of death (if applicable), and the sample types available are included. If multiple samples are available for a specific sample type, the number (n) of samples available is indicated in between brackets directly behind the specific sample type. a = EDTA whole blood, b = PBMCs, c = Heart, d = Liver, e = Tongue, f = Lung, g = Kidney. EM (*Elephas maximus*) indicates Asian elephants, LA (*Loxodonta africana*) indicates African savanna elephants.

For the analysis of cytokine responses in blood, EDTA whole blood samples or peripheral blood mononuclear cells (PBMCs) were used. Samples were taken aseptically from ear or leg veins by zoo veterinary staff for diagnostic investigations according to standard veterinary practices after which their remainders were made available for the present study. EDTA whole blood samples were transported at 4 °C and stored at -80 °C upon receipt. PBMCs were isolated from heparinized whole blood samples using Histopaque^®^-1077 (Sigma-Aldrich^®^) within eight hours after blood collection and subsequently stored at -135 °C.

Tissue samples of five different organs were selected to analyze localized cytokine responses. Based on two previous reports (17, 18), three tissues (heart, liver, and tongue) with expected high viral loads and two tissues (lung and kidney) with expected low(er) viral loads were included. Necropsies of all animals were performed by local veterinarians or veterinary pathologists; tissues were stored at -80 °C directly after collection and subsequently transported to our institute on dry ice. For each sample, approximately 500 mg tissue was homogenized into 7 ml RPMI-1640 medium (Gibco) using the ULTRA-TURRAX^®^ Tube Drive (IKA) combined with DT-20 dispersing tubes (IKA). Homogenization was performed for 50 to 100 seconds at medium speed. Homogenates were aliquoted and stored at -80 °C until further processing.

To obtain positive control mRNA for the cytokine qPCRs, PBMCs from a healthy elephant were stimulated using pokeweed mitogen (PWM-lectin; 0.05 μg per one million cells; Sigma^®^-Aldrich Chemie, Zwijndrecht, the Netherlands) for 72 hours at 37°C. Post-stimulation, the cells were resuspended in RLT buffer (Qiagen) supplemented with 0.1% β-mercaptoethanol and stored at -80 °C until further processing.

### DNA isolation and EEHV qPCRs

To determine the viral loads in all samples, DNA was extracted from whole blood samples and tissue homogenates using the DNeasy Blood & Tissue Kit (Qiagen) according to manufacturer’s instructions. Minor adaptations to the protocol were made to allow processing of tissue homogenates. Briefly, 200 µl homogenate (corresponding to approximately 15 mg tissue) was mixed with 20 μl proteinase K and 200 μl buffer AL and incubated at 56 °C for 15 minutes. Subsequently, 200 μl 96% ethanol was added to the sample, sample mixes were loaded onto the column, and DNA extraction was continued as per manufacturer’s instructions.

EEHV and TNF qPCRs, the latter used to detect elephant genomic DNA, were performed using primers and probes previously described by Stanton and coworkers (28, 29) in combination with the PrimeTime™ Gene Expression Master Mix (Integrated DNA Technologies). Tenfold dilutions of linearized plasmid encoding the appropriate target DNA were taken along to allow quantification. All assays were run using the LightCycler^®^ 480 Instrument (Roche), using the following cycling conditions: 10 minutes (min) at 95°C, followed by 40 cycles of denaturation for 15 seconds (s) at 95°C and 1 min at 60°C. Results obtained were expressed as viral genome equivalents (VGE) per milliliter blood (VGE/ml) or normalized using the elephant genome equivalents (EGE), as determined by the TNF genomic DNA qPCR, as a parameter for the number of cells analyzed and expressed as viral genome equivalent per elephant genome equivalent (VGE/EGE).

### mRNA isolation and cDNA generation for cytokine assessment

mRNA was isolated from EDTA whole blood using the FavorPrep™ Blood/Cultured Cell Total RNA Purification Mini Kit (Favorgen Biotech) according to manufacturer’s instructions. For mRNA extraction from PBMCs and tissue homogenates, the RNeasy Mini Kit (QIAGEN) was used as per manufacturer’s instructions, with a few adaptations to the protocol to allow processing of tissue homogenates. Briefly, 200 µl homogenate was mixed 25 μl proteinase K and 200 μl buffer AL and incubated at 56 °C for 15 minutes. Subsequently, 200 μl 96% ethanol was added per sample, sample mixes were loaded onto the column, and RNA extraction was continued as per manufacturer’s protocol. For all sample types, an on-column DNase digestion was performed using RNase free DNase (QIAGEN). The concentration and purity of all samples were checked by spectrophotometry, after which synthesis of complementary deoxyribonucleic acid (cDNA) was performed using the iScript™ cDNA synthesis Kit (Bio-Rad) using up to 200 ng mRNA per reaction.

### Cytokine qPCRs

The primer combinations used to amplify eight different cytokines and the β-actin housekeeping gene (HKG) are shown in **Table 2**. For five targets (β-actin, TNF-α, IFN-γ, IL-4, and TGF-β), primer combinations previously described in literature (30–32) were used. For the other four targets (IL-2, IL-6, IL-10 and IL-12), novel primers were designed based on target-specific mRNA sequences available in GenBank using the online NCBI Primer-BLAST tool to ensure sensitive and specific amplification with an RT-qPCR efficiency above 95%. PCR product sizes were specified as 80 to 150 base pairs and primer melting temperatures (Tm) were set to 55 to 65 °C with an optimum of 60 °C and a maximum Tm difference of 4 °C.

**Table 2:** Overview of the primer combinations used in the study.

Target	Function	Primer sequence (5’→3’)	Reference	Assay efficiency (% ± SEM)
β-actin	Housekeeping gene	F: GGCAGGTCATCACCATTGG	(30)	102,2 (± 3,1)
		R: ACAGGATTCCATTCCCAGGAA		
IL-2	T-cell proliferation	F: CATGCCTAAGGAGGTCACAGA	This study	95,5 (± 4,1)
		R: CCTGGTGTTTTGTTTGCTTGGA		
IL-6	Pro-inflammatory	F: AGGCAAACGTCATAGTTGT	This study	99,2 (± 0,7)
		R: CGTCTTCTGGATTCTTTATCT		
TNF-α	Pro-inflammatory	F: GAGATCCAAGTGACAAGCCTGTAG	(30)	100,2 (± 1,8)
		R: TGAAGTTGCCCCTCGGTTT		
IFN-γ	Pro-inflammatory, Th1 response	F: GGAATATCTTAATGCAACTGATTCA	(31)	104,9 (± 5,8)
		R: CCTGGTTGTCTTTCAAGTTGTCAA		
IL-12	Th1 response	F: ATTAACCGCGAATGAGAATTGC	This study	104,2 (± 4,1)
		R: TGGTACATCTTCAGGTCCTCGTA		
IL-4	Th2 response	F: CAGGTCTCTAAACGCCACGA	(32)	103,4 (± 7,2)
		R: CCAGGTTTGTCATGCTGCTG		
IL-10	Anti-inflammatory, Th2 response	F: CACCTACTTCCCAGGCAGC	This study	95,3 (± 4,0)
		R: ACCCTTAAAGTCCTCCAGCAAG		
TGF-β	Anti-inflammatory	F: CGCCTGCTGAGGCAAAGT	(30)	97,9 (± 4,4)
		R: GAGGTAGCGCCAGGAATCATT		

Per target, the main (immune) function, the forward and reverse primer sequences used, a reference to the publication in which the primers were first described (if applicable), and assay efficiency are provided. F, forward; R, reverse; SEM, standard error of the mean.

Targets were amplified separately in a 25 µl PCR reaction mix containing 5 µl of cDNA (diluted 2× in nuclease-free water), 12.5 µl iTAQ™ Universal SYBR^®^ Green Supermix (Bio-Rad), 1 μl of each forward and reverse primer (final concentration of 400 nM), and nuclease-free water. Assays were run on the Bio-Rad CFX Connect™ (Bio-Rad Laboratories, Hercules, CA, USA), using the following cycling conditions: initial denaturation for 5 minutes (min) at 95°C, 40 cycles of amplification consisting of 10 seconds (s) at 95°C, 10 s at 58°C and 30 s at 72°C, denaturation for 1 min at 95°C and a melt curve analysis consisting of 1 min at 65°C, followed by 1°C increments all lasting 1 min until 95°C. All samples were assayed in duplicate and considered positive for a target if the average cycle threshold (Ct) of the duplicates was below 40 and single peaks at the expected temperature was observed upon melt curve analysis. Replicates for which no target amplification was detected (Ct > 40) were assigned a Ct value of 41.

Assay efficiency (%E) was determined by testing two-fold serial dilutions of positive control cDNA followed by simple linear regression analysis of obtained Ct values. For all primer combinations, reproducible efficiencies between 90 and 110% were detected (**Table 2**). Specificity of all assays was verified by melt curve analysis and agarose gel electrophoresis of the produced amplicon.

Results of all study samples were processed using the 2^-ΔΔCT^ method (33) and subsequently logarithmically (LOG_10_) transformed to enable statistical analysis. Samples for which no target amplification could be detected (Ct > 40) were assigned a ΔCt value of 41 minus the average β-actin (HKG) Ct value of the appropriate tissue type in order to ensure that variations in β-actin mRNA levels minimally affected statistical analyses. Despite assay optimization, a few instances of aspecific amplification were noted upon testing the eventual study samples, and these data points were excluded from further analysis.

### Statistical analyses

Statistical significance was assessed by two-way ANOVA and Mann Whitney U test. Additionally, principal component analysis (PCA) and simple linear regressions were performed to assess the correlation between viral loads and cytokine expression levels. All analyses were performed in GraphPad Prism using the recommended settings. *P* values below 0.05 were considered significant.

## Results

### Viral loads in blood and tissue samples of EEHV-HD cases and controls

In order to get more insight into the cohort used in the study, we determined the viral loads in all available samples. **Table 3** lists the viral loads detected in whole blood samples of EEHV-HD cases and control animals (non-HD). Clear viral loads (ranging from 1,61×10^4 to 2,90×10^7 VGEs/ml) were detected for all EEHV-HD cases while in none of the control animals viremia was detected. Viral loads detected for EEHV-HD survivors (n=2) appeared slightly lower than those detected for fatalities (n=5), however this could be due to the fact that survivors were generally sampled at later time points post onset of disease than fatalities and that overall viral loads were observed to decrease over time (**Supplementary Figure 1**).

**Table 3:** Viral loads in whole blood samples of EEHV-HD cases and controls.

Animal #	Age (y)	Elephant species	HD status	Days post onset of disease	Viral load	
					VGE/ml	VGE/EGE
11	2,2	EM	Fatal HD (1A)	7	1,60E+05	2,38E-02
13	2,9	EM	Fatal HD (1A)	0	2,90E+07	1,24E+00
14a	3,0	EM	Fatal HD (1A)	0	1,69E+07	1,61E+00
14b	3,0	EM	Fatal HD (1A)	1	NA	NA
15c	3,6	EM	Fatal HD (1B)	0	1,45E+07	2,00E+00
15d	3,6	EM	Fatal HD (1B)	1	1,15E+07	1,22E+00
16	4,1	EM	Fatal HD (5)	6	2,34E+06	6,38E-01
7a	1,7	EM	HD survivor (1A)	1	1,52E+06	7,68E-02
7b	1,7	EM	HD survivor (1A)	7	7,23E+05	1,71E-02
7c	1,7	EM	HD survivor (1A)	12	1,01E+05	3,29E-03
8a	4,9	EM	HD survivor (1B)	4	2,61E+05	3,46E-03
8b	4,9	EM	HD survivor (1B)	14	1,61E+04	3,28E-04
1a	0,8	EM	Non-HD	-	0,00E+00	0,00E+00
2	1,3	EM	Non-HD	-	0,00E+00	0,00E+00
1b	1,5	EM	Non-HD	-	0,00E+00	0,00E+00
1c	1,5	EM	Non-HD	-	0,00E+00	0,00E+00
15a	2,5	EM	Non-HD	-	0,00E+00	0,00E+00
15b	3,0	EM	Non-HD	-	0,00E+00	0,00E+00
3	4,2	EM	Non-HD	-	0,00E+00	0,00E+00
4	8,2	EM	Non-HD	-	0,00E+00	0,00E+00
5	10,6	EM	Non-HD	-	0,00E+00	0,00E+00
6a	21,2	EM	Non-HD	-	NA	NA
6b	22,8	EM	Non-HD	-	NA	NA

Viral loads are expressed as viral genome equivalents per milliliter whole blood (VGE/ml) or viral genome equivalents per elephant genome equivalent (VGE/EGE). For visual purposes, viral loads were assigned a color ranging from deep red for the highest viral loads to white for the intermediate viral loads and deep blue for the lowest viral loads. EM (*Elephas maximus*) indicates Asian elephants.

The viral loads detected in the sampled organs (heart, liver, tongue, lung and kidney) of EEHV-HD fatalities are shown in **Table 4**. Generally, the highest viral loads were observed in heart and liver (median VGE/EGE of 37.4 and 39.8, respectively). Viral loads detected in tongue (median VGE/EGE of 1.8) but also blood (median VGE/EGE of 1.2; **Table 3**) were more than twentyfold lower than the viral loads detected in heart and liver, and the lowest viral loads were detected in lung (median VGE/EGE of 0.11) and kidney (median VGE/EGE of 0.02). Viral loads clearly differed between cases, with relatively low viral loads detected in all organs of case 11 and 22, and exceptionally high viral loads detected in the liver of case 12. Case 12 also showed the highest viral loads in heart and tongue. While all EEHV1 cases showed comparable or higher viral loads in liver as compared to heart, the two animals that succumbed to a different EEHV species than EEHV1 (case 16 (EEHV5) and 22 (EEHV6)) had >94-fold lower viral loads in liver than in heart.

**Table 4:** Viral loads in tissues of eight EEHV-HD fatalities.

Animal #	Age (y)	Elephant species	EEHV (sub)species	Heart	Liver	Tongue	Lung	Kidney
9	1,1	EM	EEHV1A	NA	3,56E+01	3,19E+00	NA	NA
10	1,4	EM	EEHV1A	NA	5,59E+01	NA	NA	6,91E-02
11	2,2	EM	EEHV1A	4,32E-02	1,94E-01	6,64E-02	6,46E-02	2,08E-03
12	2,6	EM	EEHV1A	2,35E+02	1,39E+04	1,12E+01	3,07E-01	3,23E-02
14	3,0	EM	EEHV1A	4,11E+01	8,31E+01	1,27E+00	1,38E-01	1,05E-02
15	3,6	EM	EEHV1B	3,36E+01	4,40E+01	2,40E+00	3,08E-01	1,89E-02
16	4,1	EM	EEHV5	4,52E+01	3,04E-01	2,25E-01	8,95E-02	2,56E-02
22	2,0	LA	EEHV6	1,75E-01	1,85E-03	NA	2,24E-03	2,14E-03

Data are presented as in **Table 3**. Only the viral loads for the EEHV species causing disease are shown in VGE/EGE. EM (*Elephas maximus*) indicates Asian elephants. LA (*Loxodonta africana*) indicated African savanna elephants.

Also for four out of six control animals, at least one EEHV species could be detected in at least one of the sampled organs (**Table 5**), yet viral loads were much lower than those detected for the EEHV-HD fatalities. Since these animals died from other causes than EEHV-HD and viral loads were relatively low and potentially reflected latent infections with EEHV or reactivation thereof, we considered it acceptable to use these tissues as control samples in the study.

**Table 5:** Viral loads detected in tissues of the controls.

Animal #	Age (y)	Elephant species	EEHV species	Heart	Liver	Tongue	Lung	Kidney
23	0,01	LA	EEHV2	0,00E+00	0,00E+00	NA	0,00E+00	0,00E+00
			EEHV3/7	0,00E+00	0,00E+00	NA	0,00E+00	0,00E+00
			EEHV6	0,00E+00	0,00E+00	NA	0,00E+00	0,00E+00
17	0,05	EM	EEHV1	NA	7,98E-05	NA	NA	NA
			EEHV4	NA	0,00E+00	NA	NA	NA
			EEHV5	NA	0,00E+00	NA	NA	NA
18	0,91	EM	EEHV1	0,00E+00	0,00E+00	0,00E+00	0,00E+00	0,00E+00
			EEHV4	0,00E+00	0,00E+00	0,00E+00	0,00E+00	0,00E+00
			EEHV5	0,00E+00	0,00E+00	0,00E+00	0,00E+00	0,00E+00
19	9,10	EM	EEHV1	0,00E+00	0,00E+00	7,19E-03	5,75E-04	0,00E+00
			EEHV4	0,00E+00	0,00E+00	0,00E+00	0,00E+00	0,00E+00
			EEHV5	0,00E+00	0,00E+00	0,00E+00	0,00E+00	0,00E+00
20	39,93	EM	EEHV1	0,00E+00	0,00E+00	0,00E+00	0,00E+00	0,00E+00
			EEHV4	0,00E+00	0,00E+00	0,00E+00	0,00E+00	0,00E+00
			EEHV5	2,65E-05	0,00E+00	0,00E+00	0,00E+00	0,00E+00
21	53,02	EM	EEHV1	0,00E+00	1,10E-05	0,00E+00	0,00E+00	0,00E+00
			EEHV4	0,00E+00	0,00E+00	0,00E+00	0,00E+00	0,00E+00
			EEHV5	0,00E+00	0,00E+00	0,00E+00	0,00E+00	0,00E+00

Data is presented as in **Table 4**. Viral loads of all EEHV species relevant to the respective elephant species are shown in VGE/EGE.

### Comparison of the cytokine response in blood samples of EEHV-HD cases and controls

**Figure 1:**
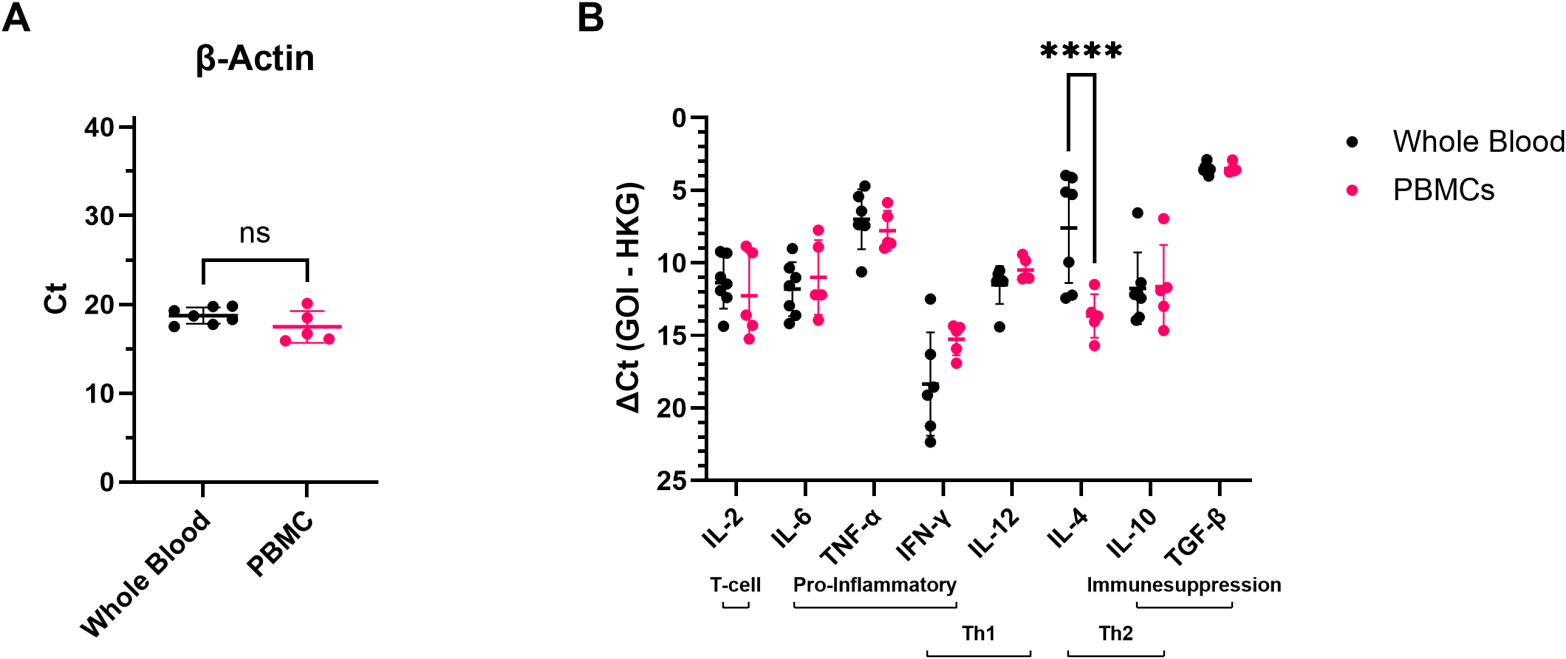
mRNA levels of the different targets detected in EDTA whole blood or PBMCs of healthy control elephants. **(A)** The cycle threshold (Ct) values obtained for the housekeeping gene β-actin in EDTA whole blood and PBMC samples. Significance was assessed by Mann Whitney U test using GraphPad Prism. ns = not significant. **(B)** mRNA levels detected for the different cytokines expressed as ΔCt values (Ct value obtained for the gene of interest (GOI) minus the Ct value obtained for the housekeeping gene (HKG)). Significance was assessed by two-way ANOVA including Šídák’s multiple comparisons test using GraphPad Prism, and only significant differences are indicated. **** indicates p < 0.0001.

Because all blood samples used in this study were remainders of samples taken for diagnostic purposes, two different sample types (EDTA whole blood samples or isolated PBMCs; **Table 1**) were available to examine cytokine responses in blood cells. To verify that the detected cytokine mRNA levels were comparable across both sample types, (Δ)Ct values obtained for EDTA whole blood samples and PBMCs of healthy controls were compared (**Figure 1**). Expression levels of the housekeeping gene β-actin (**Figure 1A**) and most cytokines (**Figure 1B**) were comparable across both sample types. For IL-4, however, significantly higher mRNA levels were detected in EDTA whole blood samples than in PBMCs (**Figure 1B**), which is in line with the fact that granulocytes, major producers of IL-4 (34), are present in whole blood but not in PBMCs. Based on these results, it was decided to combine the data obtained for EDTA whole blood samples and PBMCs for all further cytokine expression analyses, except for IL-4, for which analyses were performed separately.

**Figure 2:**
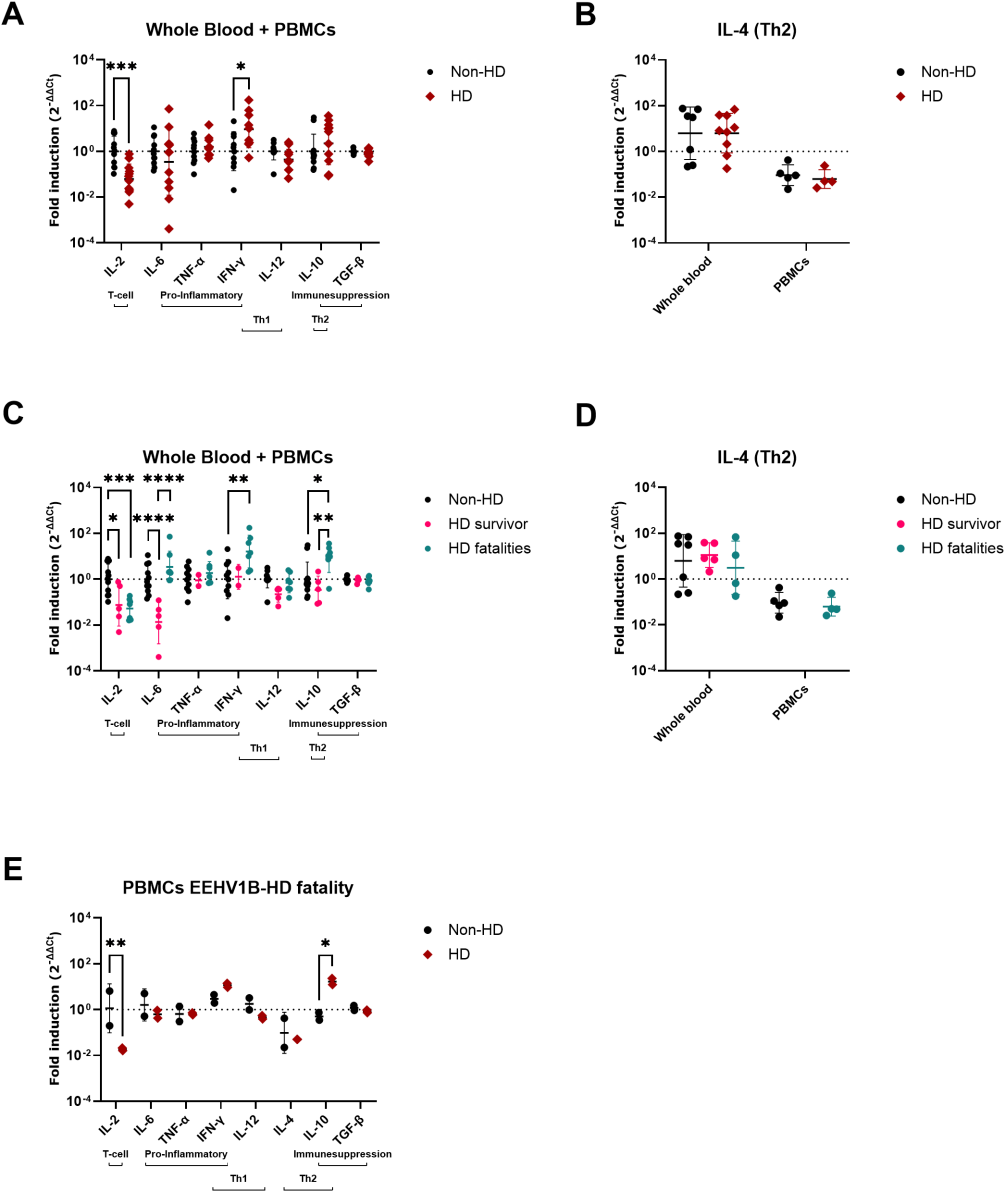
Cytokine expression levels in blood samples of EEHV-HD cases as compared to control elephants. The fold inductions shown were determined using the 2^-ΔΔCt^ method (33) and LOG_10_ transformed to enable statistical analysis. **(A, B)** Differences in cytokine expression levels between control (non-HD) and EEHV-HD (HD) elephants. **(B)** For IL-4, analyses between control elephants and EEHV-HD cases were performed separately for EDTA whole blood samples and PBMCs. **(C, D)** Differences in cytokine expression levels between control elephants, EEHV-HD survivors, and EEHV-HD fatalities. **(D)** For IL-4, analyses between controls, survivors and fatalities were performed separately for EDTA whole blood samples and PBMCs. PBMCs were not available for the EEHV-HD survivors. **(E)** Changes in cytokine expression levels in 4 longitudinal PBMC samples of one fatal EEHV1B-HD case. The non-HD samples were taken approximately 12 and 6 months before its fatal EEHV-HD episode, while the HD samples were taken 1 day before death and perimortem. In all panels significance was assessed by two-way ANOVA including Šídák’s multiple comparisons test using GraphPad Prism and only significant differences are indicated: * indicates *p* < 0.05, ** indicates *p* < 0.01, *** indicates *p* < 0.001 and **** indicates *p* < 0.0001.

The comparison of cytokine expression levels between all EEHV-HD cases (survivors and fatalities combined) and controls (**Figures 2A, B**) revealed that EEHV-HD cases had significantly decreased expression of IL-2 and increased expression of IFN-γ compared to the control animals. When the EEHV-HD cohort was further subdivided into fatalities and survivors, both groups showed significantly decreased IL-2 expression compared to the control animals, however only the fatalities showed increased expression of IFN-γ (**Figures 2C, D**). Additionally, the fatalities showed significantly increased IL-10 expression compared to both controls and survivors. Finally, the survivors showed significantly lower IL-6 expression levels compared to both the fatalities and controls, yet no significant difference in IL-6 levels was observed between the fatalities and controls.

For case 15, which succumbed to EEHV1B-HD at 3.5 years of age, PBMCs were available from two time points before (collected at 2.5 and 3 years of age) and during disease (2 samples; collected upon onset of disease and peri-mortem). Largely in line with the cross-sectional comparison between fatalities and controls (**Figure 2C**), a significant decrease in IL-2 and increase in IL-10 expression levels were observed during the EEHV-HD episode of this animal (**Figure 2E**).

**Figure 3:**
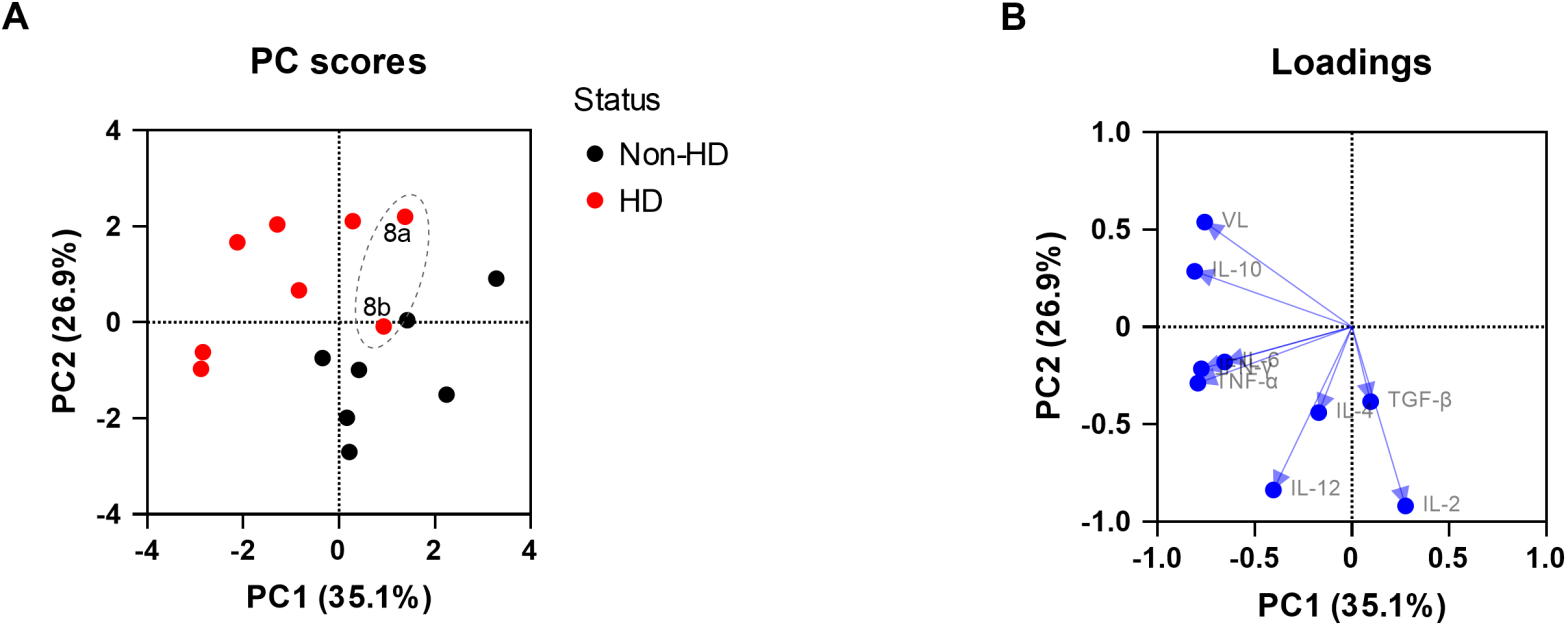
Principal component analysis showing the correlations between EEHV-HD, viral loads, and cytokine expression levels in blood cells. Both the PC scores **(A)** and loadings **(B)** plot are shown. In panel A, the samples of the only EEHV-HD survivor analyzed (case #8) are annotated and circled by a dashed grey line.

To explore the relationship between viral loads, the cytokine expression levels, and EEHV-HD further, a PCA was performed. A clear separation between the EEHV-HD cases and the control animals could be visualized based on the first two principal components, which together explained 62% of the variance (**Figure 3A**). In general, the EEHV-HD cases clustered towards the left upper quadrant of the graph, whereas most controls were found in the right lower quadrant of the graph. Notably, the two samples from the only EEHV-HD survivor that could be included in this analysis (case #8) clustered more towards the samples of the control animals than those of the EEHV-HD fatalities. This was especially true for sample 8b, which was taken 14 days after onset of disease. We believe this clustering suggests that, even though still viremic, the animal had already largely recovered from disease 14 days post onset of clinical signs.

Upon analysis of the PCA loading scores both viral load (VL) and IL-10 levels were found to be clearly correlated with each other and EEHV-HD (**Figure 3B**). In contrast, IL-2 was strongly skewed toward the quadrant opposite to where most EEHV-HD cases cluster, suggesting an inverse correlation. Like the majority of the EEHV-HD fatalities, TNF-α, IFN-γ, and IL-6 levels had highly negative PC1 values, suggesting an association between these factors and EEHV-HD. No association between EEHV-HD and the expression levels of IL-4, IL-12 and TGF-β was observed.

### Comparison of cytokine responses in tissues of EEHV-HD fatalities and controls

**Figure 4:**
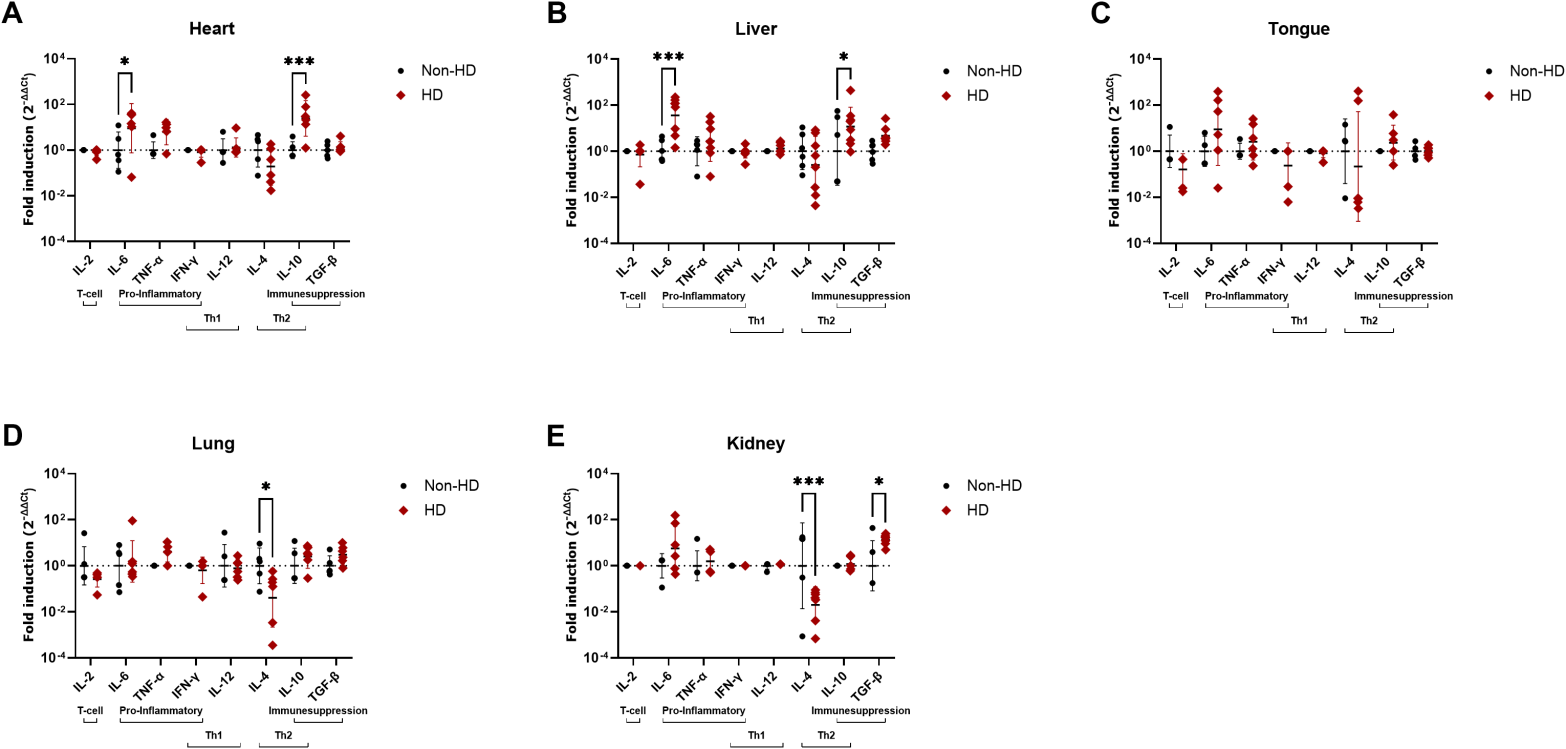
Cytokine expression levels in five different tissues of EEHV-HD cases as compared to control elephants. Results are shown separately for heart **(A)**, liver **(B)**, tongue **(C)**, lung **(D)** and kidney **(E)**. The fold inductions shown were determined using the 2^-ΔΔCt^ method and LOG_10_ transformed to enable statistical analysis. Significance was assessed by two-way ANOVA including Šídák’s multiple comparisons test using GraphPad Prism and only significant differences are indicated: * indicates *p* < 0.05 and *** indicates *p* < 0.001.

In heart and liver, the organs in which the highest viral loads were detected (**Table 4**), both IL-6 and IL-10 expression levels were significantly elevated in EEHV-HD cases as compared to controls (**Figures 4A, B**). In tongue, a tissue in which moderate viral loads were detected (**Table 4**), no significant differences in cytokine expression levels were found between cases and controls (**Figure 4C**). Finally, in lung and kidney, the organs in which the lowest viral loads were detected, significantly decreased IL-4 levels were detected for cases as compared to controls (**Figures 4D, E**). In addition, significantly increased TGF-β expression levels were detected in the kidneys of cases as compared to controls (**Figure 4E**).

**Figure 5:**
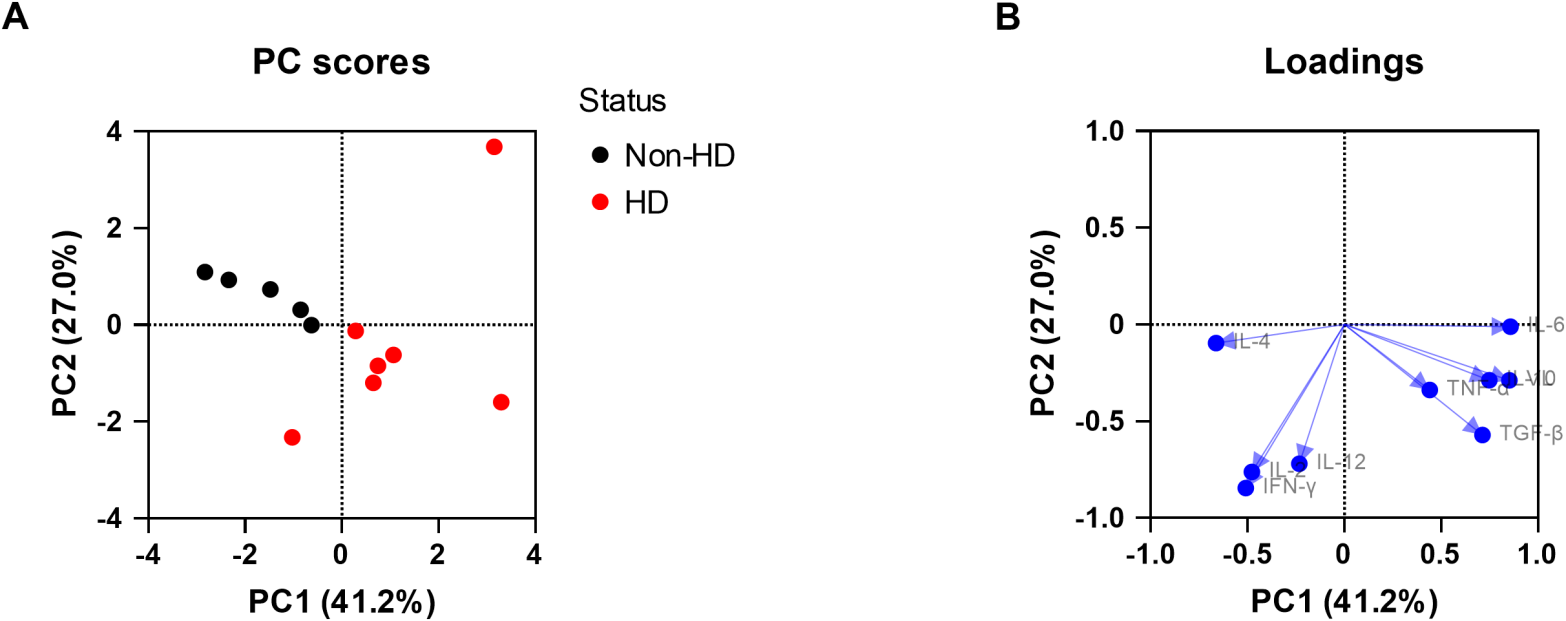
Principal component analysis (PCA) showing the correlations between EEHV-HD, viral loads, and cytokine expression levels in liver. Both the PC scores **(A)** and loadings **(B)** plot are shown.

Similar to the blood samples, we attempted to perform PCAs for all tissues analyzed with the aim to explore the relationship between viral loads, the cytokine expression levels and EEHV-HD. For liver, PCA could be successfully performed and clear separation between the EEHV-HD cases and the control animals could be observed based on the first two principal components, which together explained 68% of the variance (**Figure 5A**). The EEHV-HD cases and the controls seemed to be primarily separated alongside the PC1 axis, with EEHV-HD being strongly associated with IL-6, VL, IL-10, TGF-β and to a lesser extent TNF-α (**Figure 5B**). Additionally, we observed a moderate inverse correlation between IL-4 and the EEHV-HD cases. The fact that the loadings for IL-2, IFN-γ and IL-12 were positioned perpendicular to viral load and thereby the EEHV-HD cases, suggests these cytokines are not associated with EEHV-HD in this tissue.

**Figure 6:**
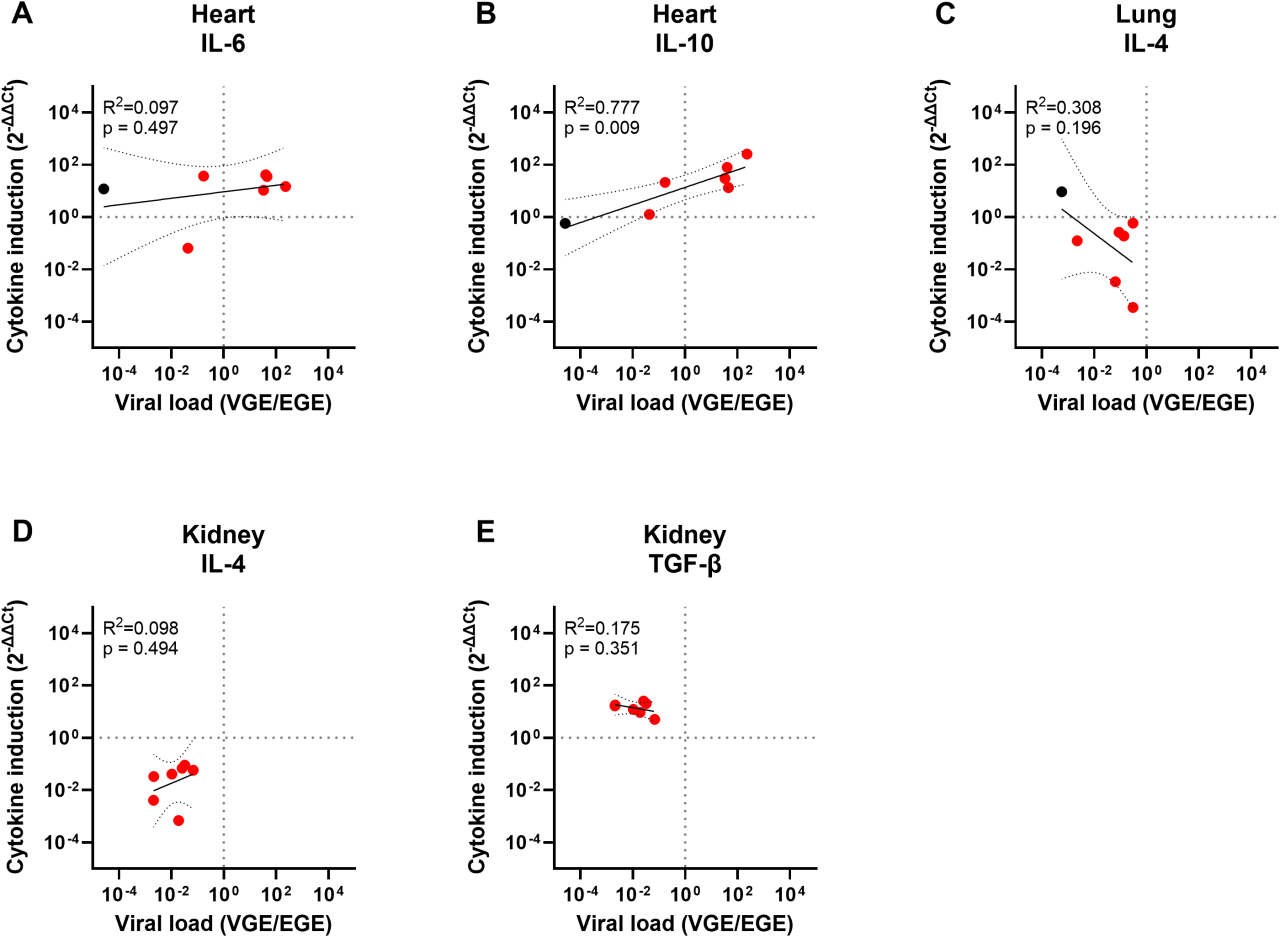
Simple linear regression analyses of the significantly altered cytokine levels and viral loads in heart, lung or kidney. Panel **(A)** shows the pairwise linear regression of the IL-6 induction levels and viral load in heart, **(B)** IL-10 induction and viral load in heart, **(C)** IL-4 induction and viral load in lung, **(D)** IL-4 induction and viral load in kidney and **(E)** TGF-β induction and viral load in kidney. Regression analyses were only performed between the cytokine expression levels found to be significantly altered in **Figures 4A, D, E** and viral loads previously shown in **Table 4** and **5**. Both the cytokine induction levels and the viral loads were LOG_10_ transformed to enable analysis. R^2^ and p-values calculated for each regression are shown in the top left corner of each panel. Data points for EEHV-HD cases are shown in red and data points for controls are shown in black. Only controls with a detectable viral load could be included.

For the other tissues, only one (heart) or no principal component (tongue, lung and kidney) could be selected, preventing PC analysis. Therefore, we assessed the correlation between viral load and the significantly altered cytokine levels by simple linear regression. In heart, a strong and significant correlation was found between VL and IL-10 induction, yet no correlation between VL and IL-6 induction could be observed (**Figures 6A, B**). Additionally, no linear correlations could be detected between the viral loads and the significantly altered cytokine expression levels in lung or kidney (IL-4 in lung and IL-4 and TGF-β in kidney; **Figures 6C–E**). These results are in line with the fact that no principal component met the criteria for selection for tongue, lung, and kidney and suggests that the variables in these data sets are nearly linearly independent.

## Discussion

The current study served to assess whether an excessive (pro-inflammatory) cytokine response, a so-called ‘cytokine storm’, contributes to EEHV-HD pathogenesis. Significant changes in mRNA expression levels of several cytokines were detected in samples from 11 EEHV-HD cases as compared to those of 12 control animals. Specifically, IL-2 levels were decreased in blood of both EEHV-HD fatalities and survivors, while the levels of IFN-γ and IL-10 were increased in blood of the EEHV-HD fatalities. PCA indicated that IL-10 levels and viral loads in blood were highly correlated with each other and EEHV-HD. Additionally, a link between IFN-γ, TNF-α and IL-6 levels in blood and EEHV-HD was shown. Finally, in liver and heart, the two tissues with the highest viral loads, significantly increased levels of both IL-6 and IL-10 were found for the EEHV-HD fatalities as compared to controls. The induction of both cytokines was positively associated with the viral load in liver, and additionally a strong and significant correlation between viral load and IL-10 induction was detected in heart. Overall, the current study confirms the presence of significantly elevated mRNA levels of cytokines typically associated with pathogen-induced cytokine storms (IFN-γ, IL-6 and IL-10) (22–26) in elephants that succumbed to EEHV-HD. Occurrence of acute systemic inflammation and multiple organ dysfunction, two other common characteristics of a cytokine storm in the context of infection, had already been reported previously by three independent publications (14–16). Based on the relatively limited viral load and the lack of clearly detectable immune activation in tissues other than heart and liver (**Table 4**, **Figures 4C–E**), any pathological changes observed in these tissues could be considered collateral damage and beyond a normal response to infection. In conclusion, we propose that the immunopathology observed during EEHV-HD indeed meets the three criteria of a cytokine storm in the context of infection (22), as described in the introduction.

While the conclusion that a cytokine storm appears to underlie EEHV-HD pathology seems similar to the conclusion drawn previously by Guntawang and coworkers (15), the exact cytokines found to be elevated clearly differed between both studies. Guntawang and coworkers assessed the induction of six different cytokines (IL-1β, IL-2, IL-4, IL-8, IFN-γ and TNF-α) in heart, liver, and lung of EEHV1-HD and EEHV4-HD fatalities separately. While all cytokines were found to be elevated in at least one of the tissue/EEHV-HD combinations tested, overall the most prominent induction was observed for IL-2, IL-4 and IFN-γ. Notably, none of these cytokines were found to be significantly elevated in heart, liver or lung in our study population (**Figures 4A, B, D**). Instead, for IL-4, we detected lower expression levels in lung tissues of EEHV-HD cases as compared to controls (**Figure 4D**). The discrepancies between the results of both studies may have been caused by the use of different primer combinations to detect cytokine mRNAs. The primers for the detection of IL-2, IL-4 and IFN-γ as used by Guntawang and coworkers were originally developed by Landolfi et al. (30) to be used in combination with a sequence-specific probe in a TaqMan qPCR format (15, 30). In the past we also tested those specific primer combinations in a SYBR Green qPCR format, both used in the current study and by Guntawang and coworkers (15), but in our hands these combinations did not meet the requirements on assay efficiency and specificity (as described in materials and methods). Therefore, we selected alternative primer combinations for these targets that did meet the requirements (**Table 2**). The two cytokines for which we detected elevated expression levels in heart and liver samples (IL-6 and IL-10), were not included in the study of Guntawang and coworkers and therefore results on these cytokines cannot be compared.

The other study that previously addressed cytokine levels during EEHV-HD, compared serum levels of IL-2 and TNF-α between elephants with and without EEHV viremia, but detected no differences between both groups (27). Based on these results and the fact that only low levels of TNFα and IL-2 were detected in serum of EEHV-HD fatalities, Edwards and coworkers argued against the hypothesis that a cytokine storm underlies EEHV-HD. Since not all animals with EEHV viremia develop EEHV-HD, it is difficult to relate the data published by Edwards and coworkers to our study. However, the lack of induction of both IL-2 and TNF-α are in line with the results presented in this study. Additionally, the observation that peak IL-2 concentrations were significantly lower in EEHV-HD fatalities compared to the other viremic animals, as reported by Edwards and coworkers, appears to be in line with the decreased IL-2 mRNA levels in EEHV-HD cases observed in the current study.

Especially IL-6, an important mediator of the acute inflammatory response, seems to play a crucial role in different types of cytokine storms (23). For the EEHV-HD fatalities in the current study, a (median) 25-fold and 100-fold IL-6 induction was detected in heart and liver, respectively. Similar fold inductions of IL-6 were previously associated with cytokine storms induced by COVID-19 infections in humans (35–38). We propose that the elevated IL-6 levels detected in EEHV-HD cases may largely explain the pathophysiological features observed during EEHV-HD. For example, IL-6 can directly and indirectly cause injury to the vascular endothelium and trigger activation of the coagulation cascade, leading to the vascular leakage and enhanced intravascular clot formation (23), two hallmarks of EEHV-HD pathogenesis (14–16). Activation of the coagulation cascade in turn leads to increased production of additional proinflammatory cytokines, including IL-6, which further amplifies this cascade (23). Additionally, elevated IL-6 levels can trigger neutrophil migration by inducing production of chemokines like IL-8 and MCP-1 and increased expression of adhesion molecules on endothelial cells (39). Locally, these neutrophils may produce neutrophil extracellular traps (NETs), a network of fibers that contributes to thrombi formation and again amplifies cytokine production (22). Influx of heterophils, the elephant equivalent of neutrophils, the presence of NETs, and formation of microthrombi have all been reported in tissues of EEHV-HD cases (14–16, 40), suggesting this cascade may indeed be triggered during EEHV-HD. Finally, elevated levels of IL-6 may lead to diminished cytolytic function of NK cells, resulting in prolonged antigenic stimulation and difficulty resolving inflammation (22). Although the role of NK cells in controlling EEHV infections has not been studied to date, this cell type is known to play an important role in immunity against many herpesviruses (41, 42).

While both IL-6 and IFN-γ are proinflammatory cytokines that can contribute to the systemic inflammation occurring during cytokine storms, the increased levels of the immunomodulatory cytokine IL-10 detected in blood, heart, and liver and the decreased levels of the T cell stimulatory cytokine IL-2 in blood of EEHV-HD cases may appear to contradict the occurrence of a cytokine storm, as these are immune dampening by themselves. Elevated levels of IL-10 levels are however commonly detected in cytokine storms and presumably reflect the regulatory mechanism to control the induction of the proinflammatory cytokines (22). In addition, decreased peripheral T cell levels have previously been reported during cytokine storms (25), and potentially reflect the sequestration of T cells into affected tissues. Since T cells are the primary source of IL-2, we hypothesize that migration of EEHV-specific T cells into affected tissues may at least in part explain the decreased IL-2 expression in blood observed in the current study. This hypothesis is supported by the fact that IL-2 expression, albeit at low levels, could occasionally be detected in heart and liver of EEHV-HD cases (25% and 33% of the samples tested, respectively), but was not detected in heart or liver of the control animals. Nevertheless, we hypothesize that that the decreased IL-2 mRNA level in blood cells, as observed for all EEHV-HD cases in this study, may affect T cell proliferation in the blood and lymph nodes overall and thus the strength of the adaptive immune response in these animals in a negative manner, thereby potentially delaying viral clearance.

Induction of the proinflammatory cytokines IFN-γ and IL-6 was either detected in blood or tissues with high viral loads (heart and liver), whereas the immunomodulatory cytokine IL-10 was found to be induced in all three aforementioned sample types. The observation that pro-inflammatory cytokine induction was specific for blood or tissues with high viral loads (heart and liver) may appear unexpected and could raise the question to what extent the detected cytokines, especially the local IL-6 induction, contribute to the systemic inflammation as observed during EEHV-HD (14–16). We hypothesize that the detection of IL-6 or IFN-γ is dependent on the cell types producing these cytokines and could also be influenced by whether these cells are infected or not. For example, endothelial cells, the major target cell of EEHV *in vivo* (19), readily produce IL-6 as a response to pathogen associated molecular patterns, detection of specific cytokines (e.g. IL-1β or IL-6), or the presence of coagulation factors (23) and may thus be the source of the high levels of IL-6 detected in heart and liver. In contrast, the mRNA transcripts detected in blood most likely reflect the activation of non-infected immune cells. Even though IL-6 was not found to be significantly induced in blood, it is important to note that PCA did reveal a link between IL-6 induction, IFN-γ induction and EEHV-HD in this sample type (**Figure 3**). Additionally, next to the cytokines produced directly in blood, IL-6 produced by endothelial cells lining the blood vessels is likely secreted into the blood and could potentially have systemic effects. Whether elevated IL-6 levels are indeed present in serum of EEHV-HD cases should be determined by a validated elephant IL-6 ELISA, which is not available to date.

Next to heart, liver, and tongue, tissues in which high to moderate viral loads are present [**Table 4** and references (17, 18)], we also included two tissues with much lower viral loads [(lung and kidney, **Table 4** and references (17, 18)] to serve as controls within the study. The fact that significant inductions of IL-6 and IL-10 could only be detected in heart and liver of EEHV-HD cases, but not in the tissues with lower viral loads further strengthens our conclusion that IL-6 and IL-10 induction are directly related to viral replication. Nevertheless, also in lung and kidney significant alterations of cytokine expression levels between EEHV-HD cases and controls were observed. Specifically, decreased IL-4 levels were detected in both lung and kidney and increased TGF-β levels were observed in kidney of the EEHV-HD cases (**Figures 4D, E**). Since IL-4 is an important modulator of the immune system that can help to prevent excessive inflammation (43–45), the decreased IL-4 expression levels in lung and kidney could be in line with the conclusion that a cytokine storm underlies EEHV-HD. However, no correlation was observed between the IL-4 or TGF-β expression levels and the differences in viral loads between tissues (**Figure 4**) or within the tissues in which the altered cytokine expression levels were detected (**Figures 5E–G**). Therefore, it could also be that these alterations were not directly caused by the ongoing EEHV infection, but instead reflect differences in age, cellular constitution, or presence of underlying illnesses between the cases and the controls; factors we could not control for because of the limited availability of samples.

While all EEHV species appear omnipresent in their respective elephant host, the majority of EEHV-HD fatalities are caused by EEHV1 (2). Interestingly, all EEHV1 cases within our cohort (n=6) showed high viral loads in liver, often comparable to or higher than in heart, yet the two animals that died to EEHV-HD caused by EEHV5 or EEHV6 showed almost 100-fold lower viral loads in liver than in heart. In line with the relatively low viral loads in liver, both the EEHV5- as well as the EEHV6-HD case also showed relatively low IL-6 induction as compared to most EEHV1-HD cases. These results suggest that the tissue tropism of the different EEHV species differs slightly, which would be in line with several necropsy reports (14, 46, 47). Pathological lesions in the liver and increased ALT concentrations, indicative of liver injury, have previously been described in EEHV(1)-HD cases (14, 48). Since the liver is an important site of synthesis of nearly all coagulation factors and pro-inflammatory cytokines such as IL-6 (49–51), and both pro-inflammatory cytokine induction and perturbed coagulation appear to underlie EEHV-HD, it would be interesting to determine whether the increased liver tropism of EEHV1 may contribute to its relatively high pathogenicity compared to other EEHV species.

Overall, our results suggest that cytokines, especially IL-6, play a crucial role in EEHV-HD pathogenesis. The exact trigger for the elevated cytokine levels detected during EEHV-HD remains uncertain, however, viral infection of the endothelial cells resulting in cell damage could potentially already be sufficient to induce substantial amounts of IL-6 and start a deleterious cascade leading to vascular leakage, tissue damage, and eventually death (20, 23). In analogy to other IL-6-mediated cytokine storms, usage of IL-6-specific or broadly-acting anti-inflammatory therapies, such as glucocorticoids (23, 52), may help to improve the outcome of EEHV-HD. Glucocorticoids have already been cautiously used to treat EEHV-HD, and several animals receiving (among others) this treatment survived disease (14). Also the two EEHV-HD survivors within our cohort were treated with glucocorticoids for multiple days during their EEHV-HD episode (personal communications). Markedly, both survivors had significantly decreased IL-6 mRNA levels in blood cells as compared to EEHV-HD fatalities and control animals (**Figure 2C**). Especially the difference with the control animals suggests the decrease in IL-6 expression levels could be the result of the treatment received. At least two fatalities within our cohort also received glucocorticoids during treatment, however the dosages used were lower and duration of treatment was shorter than for the survivors (personal communications). Whether intensive treatment with glucocorticoids indeed improves the outcome of EEHV-HD and at what stage of infection treatment needs to be initiated, needs to be addressed in future studies.

In conclusion, combined with the current knowledge on EEHV-HD lesions, our results suggest that an excessive pro-inflammatory immune response contributes to EEHV-HD pathogenesis and supports investigation into the use of corticosteroids for the control of EEHV-HD. Additionally, the observed connection between liver tropism and pathogenicity of EEHV1 is noteworthy and would be interesting to investigate in more detail.

## Data Availability

The original contributions presented in the study are included in the article/**Supplementary Material**. Further inquiries can be directed to the corresponding author.
