# Supplementary material for: Elevated IL-6, IL-10, and IFN-γ levels in fatal elephant endotheliotropic herpesvirus – hemorrhagic disease cases suggest an excessive proinflammatory cytokine response contributes to pathogenesis
Source: Front Immunol. 2025 Oct 27;16:1645752. doi: 10.3389/fimmu.2025.1645752 (PMC12597985; doi:10.3389/fimmu.2025.1645752)
Supplement: Supplementary file 1 [file Table1.docx]

Supplementary Material

Elevated IL-6, IL-10, and IFN-γ levels in fatal elephant endotheliotropic herpesvirus – hemorrhagic disease (EEHV-HD) cases suggest an excessive proinflammatory cytokine response contributes to pathogenesis

**Tabitha E. Hoornweg^1,2*^, Willem Schaftenaar^3^, Jooske IJzer^4^, Myrna M.P. Mulder^1^, Mariska Lugtenburg^1^, Anne van Beest^1^, Cornelis A.M. de Haan^2^, Victor P.M.G. Rutten^1,5^**

^1^Section Immunology, Division Infectious Diseases and Immunology, Department of Biomolecular Health Sciences, Faculty of Veterinary Medicine, Utrecht University, 3584 CL Utrecht, The Netherlands.

^2^Section Virology, Division Infectious Diseases and Immunology, Department of Biomolecular Health Sciences, Faculty of Veterinary Medicine, Utrecht University, 3584 CL Utrecht, The Netherlands.

^3^EAZA Elephant TAG, Rotterdam Zoo, 3041 JG Rotterdam, The Netherlands.

^4^Division Pathology, Department of Biomolecular Health Sciences, Utrecht University, Faculty of Veterinary Medicine, 3584 CL Utrecht, The Netherlands.

^5^Department of Veterinary Tropical Diseases, Faculty of Veterinary Science, University of Pretoria, Onderstepoort 0110, South Africa.

**
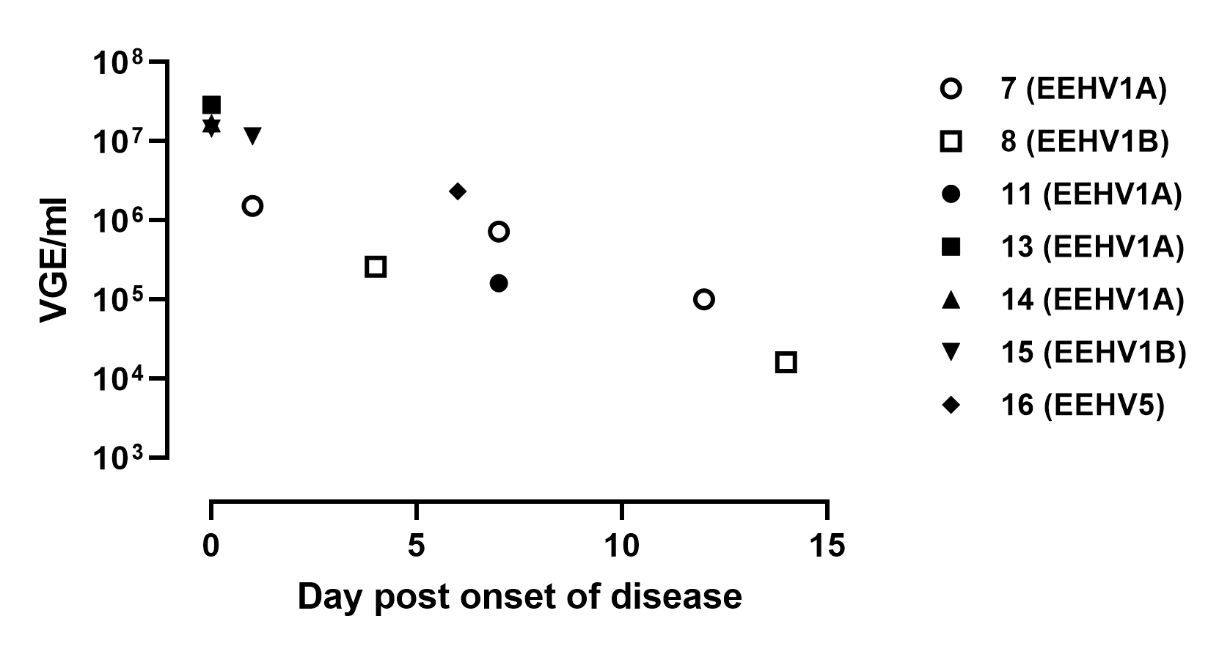
**

**Supplementary Figure 1. Viral loads detected in whole blood samples of EEHV-HD fatalities and survivors, plotted based on the number of days post onset of disease the sample was taken.** Each symbol represents an individual animal, with open symbols representing EEHV-HD survivors and closed symbols representing EEHV-HD fatalities. The subspecies that caused disease is indicated in the legend. VGE/ml = viral genome equivalents per milliliter.
